# Sanshen San Formula Hinders Cognitive Function and Pathology in Alzheimer's Disease Through Potentiating the Function of Synapse

**DOI:** 10.1111/cns.70349

**Published:** 2025-04-09

**Authors:** Shiquan Chang, Nana Ding, Yalin Li, Ying Li, Ziling Tang, Junping Pan, Li Yan, Jiaxu Chen

**Affiliations:** ^1^ Guangzhou Key Laboratory of Formula‐Pattern of Traditional Chinese Medicine, Jinan University Guangzhou China; ^2^ College of Basic Medicine Hubei University of Chinese Medicine Wuhan China; ^3^ Guangdong Second Provincial General Hospital, Postdoctoral Research Station of Basic Medicine, School of Traditional Chinese Medicine Jinan University Guangzhou China; ^4^ School of Traditional Chinese Medicine Beijing University of Chinese Medicine Beijing China

**Keywords:** Alzheimer's disease, autophagy, neuron, Sanshen san formula, Traditional Chinese medicine

## Abstract

**Background:**

Alzheimer's disease (AD) constitutes a devastating neurodegenerative disorder, manifested by amyloid‐β aggregation, phosphorylated tau accumulation, and progressive cognitive deterioration. Current therapeutic interventions remain predominantly symptomatic, underscoring the urgency for more efficacious treatment strategies.

**Purpose:**

This study elucidated the therapeutic potential of Sanshen San (SSS), a traditional Chinese herbal formula encompassing *Polygala Radix*, *Pini Radix in Poria*, and *Acori Tatarinowii Rhizoma*, on cognitive function and AD pathology.

**Methods:**

We implemented both acute Aβ_1‐42_‐injected mice and 5xFAD transgenic mouse models. The therapeutic efficacy of SSS was assessed through behavioral paradigms including Y‐maze, Novel Object Recognition, and Morris Water Maze. Molecular mechanisms were delineated utilizing RNA sequencing, metabolomics analysis, immunofluorescence staining, Golgi‐Cox staining, transmission electron microscopy, and Western blotting.

**Results:**

Chemical analysis unveiled 10 principal bioactive compounds in SSS. The formula substantially ameliorated cognitive performance in both Aβ_1‐42_‐injected and 5xFAD mouse models, attenuated Aβ plaque burden, and augmented microglial phagocytosis. SSS safeguarded synaptic integrity, enhanced mitochondrial function, and facilitated autophagy. Transcriptomic and metabolomic analyses demonstrated that SSS predominantly operates by reinstating synaptic transmission and neurotransmitter function, particularly in the dopaminergic system.

**Conclusion:**

SSS efficaciously mitigates AD pathology through potentiating synaptic function, optimizing mitochondrial homeostasis, and restoring neurotransmitter balance, exemplifying a promising multi‐target therapeutic strategy for the treatment of AD.

## Graphical abstract

SanshenSan formula efficaciously mitigates AD pathology through potentiating synaptic function, optimizing mitochondrial homeostasis, and restoring neurotransmitter balance, exemplifying a promising multi‐target therapeutic strategy for the treatment of Alzheimer's disease.

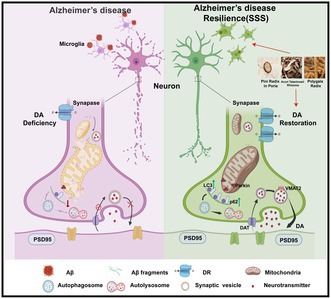

**Abbreviations**
5xFAD
: The five‐familial Alzheimer's disease transgenic mouse
AD
: Alzheimer's disease
DA: dopamine
DAPI: 4′,6‐diamidino‐2‐phenylindole
GO: gene ontology
KEGG: Kyoto encyclopedia of genes and genomes
MWM: morris water maze
NOR: novel object recognition
OPLS‐DA: orthogonal partial least squares discrimination analysis
PBS: phosphate‐buffered saline
PFA: paraformaldehyde
SSS: Sanshen San
TCM: traditional Chinese medicine
TEM: transmission electron microscopy
UHPLC–MS/MS: high‐performance liquid chromatography tandem mass spectrometry
VIP: variable importance

## 1 Introduction

AD is the leading and profoundly impactful neurodegenerative disorder affecting individuals globally. As the leading cause of dementia among the elderly, AD impacts more than 30 million individuals universally. More than 100 million people worldwide are predicted to have AD by 2050 [1]. The increasing prevalence of Alzheimer's places a heavy societal and economic burden. The condition is marked by the accumulation of amyloid‐beta (Aβ) deposits and tau protein that has undergone phosphorylation, which is coupled with a gradual deterioration in cognitive abilities and memory retention [2]. US Food and Drug Administration (FDA)‐approved prescription drugs known as galantamine, donepezil, and memantine could not halt or decelerate the progression of the illness, as these drugs only work to treat symptoms [3]. In the past years, the field of AD drug development has embarked on a new chapter, focusing on disease‐modifying treatments. This shift has been underscored by the FDA's approval of monoclonal antibodies, including lecanemab and donanemab, marking a significant advancement in therapeutic strategies [4]. There is an imperative to cultivate innovative and potent methodologies for addressing AD treatment.

A classic therapeutic target has been amyloid‐β. The hypothesis of amyloid protein accumulation in AD proposes that the amassment of Aβ serves as a pivotal factor in a series of interrelated occurrences that lead to neuronal dysfunction and subsequent cell demise [5]. This course ultimately brings about the cognitive decline characteristic of AD [6, 7]. In addition to Aβ, synaptic loss, hyperactive neuroinflammation, impaired mitochondria, etc., are also observed in AD [8]. Lately, it has been exhibited that dysfunctional autophagy is firmly connected with AD [9]. The central feature observed in the brains of individuals suffering from AD is the presence of numerous enlarged, under‐acidified autolysosomes and autophagic vesicles containing beta‐amyloid and tau [10, 11]. Mitophagy plays a fundamental role in mitochondrial homeostasis, which is necessary for maintaining efficient synaptic activity, regulating presynaptic stability—which includes support for axonal remodeling—and thus maintaining the integrity of the neuronal network [12]. To maintain protein homeostasis, neurons rely on autophagy to remove large, insoluble protein aggregates [13]. Synapses serve as crucial junctions for the transmission of information from one neuron to another. Changes in synaptic flows might prompt deviant synaptic capability, thereby disturbing typical correspondence between neurons [14].

Memories are formed through the experience‐driven alteration of synaptic connections at the cellular level [15]. Dopamine (DA) discharge from the VTA efferent pathway neural pathways happens in an expansive scope of regions such as the prefrontal cortex (PFC), nucleus accumbens (NAc), amygdala, and hippocampus [16]. Contingent upon the conditions, dopaminergic neurons are prepared for dealing with both positive and negative reinforcement learning frameworks [17, 18]. In more mature adult individuals, there is a decrease in the quantity of dopamine receptors in the hippocampal CA1 area, showing that dopamine modulation is disabled with age [19]. The activation of β‐adrenergic and D1 dopamine receptors prompts alterations in synaptic plasticity, which in turn affects memory, with various kinases and phosphatases playing a mediating role, intervened by different kinases and phosphatases [20]. Dopamine and noradrenaline mediated the modulation of novelty, reward, and emotionality to hippocampal plasticity and memory by means of catecholamine liberation in the hippocampus, facilitating the integration of proteins associated with plasticity that contribute to the stability of synaptic plasticity [21]. Subsequently, the consistency of these synaptic changes is governed.

In the cerebrum, mitochondria are central participants in neuronal movement, as neurons are weighty consumers of energy and profoundly subject to Ca^2+^ homeostasis, both core elements of the mitochondria. The intrinsic cytotoxicity of dopamine, because of its metabolism by monoamine oxidase can generate ROS, is a peculiarity of dopaminergic neurons [22]. Unoxidized DA can pass through the mitochondria and inhibit CoI in a less oxidative cellular environment. In many cells, such a gentle decrease in energy production makes a negligible difference. Nevertheless, in neuronal cells, which are profoundly reliant on energy replenishment and the balance of calcium ions (Ca^2+^) for their functionality, the inability of mitochondria to fulfill their high‐energy demands prompts their contorted action, impeded hardware, and eventually leads to unusual mental, profound, and social capabilities [23].

As these advances in understanding AD pathogenesis, effective treatments remain limited. Traditional Chinese Medicine (TCM) has shown promise in treating various neurodegenerative disorders, including AD. Sanshen San (SSS), an ancient Chinese herbal formula consisting of *Polygala Radix, Pini Radix*, *in Poria*, and *Acori Tatarinowii Rhizoma*, has been traditionally used to treat cognitive disorders. However, its potential therapeutic effects and underlying mechanisms in AD treatment remain largely unexplored. In this study, we studied and examined the effects of SSS on cognitive function in both acute Aβ_1‐42_‐injected mice and 5xFAD transgenic mice. We further explored its impact on Aβ pathology, synaptic plasticity, mitochondrial function, and autophagy. Additionally, through RNA sequencing and metabolomics analysis, we aimed to illustrate the molecular mechanisms underlying SSS's therapeutic effects, particularly focusing on neurotransmitter systems and synaptic function. Our findings may offer fresh perspectives on the development of novel remedial systems for AD treatment.

## 2 Materials and Methods

### 2.1 Preparation of Sanshen San Formula

Sanshen San Formula is made up of three traditional Chinese herbal medicines: *Polygala Radix, Pini Radix in Poria*, and *Acori Tatarinowii Rhizoma*. All ingredients of SSS used in our study were procured from Guangzhou Baozhilin Pharmacy. We blended them at the proportion of 1:1:1 and afterward boiled them two times with multiple times water. After the decoction was blended, filtered, and evaporated, the concentrated concentrate was freeze‐dried to get lyophilized powder and stored. The doses of the low‐dose, medium‐dose, and high‐dose group (SSS‐L, SSS‐M, and SSS‐H) were 2.5 g/kg, 5.0 g/kg, and 10.0 g/kg, respectively.

### 2.2 Component Analysis of SSS

We weighed 50 mg of lyophilized powder sample and dissolved it in pure methanol to 50 mL. Then we ran the component analysis of SSS utilizing a Waters Acquity ultra‐performance liquid chromatography system by using a column from Waters HSS T3 (100 × 2.1 mm, 1.8 μm). The column temperature was 40°C, and the injection volume was 2 μL. The mobile phases A and B were ultrapure water and acetonitrile, respectively, both containing 0.1% formic acid with a flow rate of 0.3 mL/min.

### 2.3 Network Pharmacology Analysis

The SMILES sequences of main SSS components were retrieved from PubChem (http://pubchem.ncbi.nlm.nih.gov). The active ingredients were entered into SwissTargetPrediction (http://www.swisstargetprediction.ch/) to anticipate the SBP targets, and the parts for which the likelihood was more noteworthy than 0.1 were selected. An ingredient–target diagram was drawn on the condition that a probability was greater than 0.2. Alzheimer's disease gene data were obtained from the DrugBank (https://go.drugbank.com), OMIM (https://omim.org), and GeneCards (https://www.genecards.org/) databases to gain access to disease targets. The DisGeNet score was set to at least 0.02, the GeneCards score to at least 1, and the false discovery rate (FDR) < 0.05 in the GEO. We used Cytoscape 3.9.1 to visualize and analyze the results.

### 2.4 Animals

Eight‐week‐old C57BL/6J mice were purchased from Charles River Laboratory Animal Technology Co. Ltd. (Guangzhou, China) 0.6‐month‐old male 5 × FAD and wild‐type male mice (approximately 30 g) with a C57BL/6J background were acquired from Cyagen Biosciences Co. Ltd. (Guangzhou, China). The animal ethics approval numbers are IACUC‐20220613‐19 and IACUC‐20230221‐05. Animals were housed in a SPF‐grade animal breeding room in Jinan University Laboratory Animal Center. Following a week of acclimatized nourishment, mice were categorized into four or five distinct groups utilizing a random number generator: control group(sham‐operated), AD group, AD + SSS low‐dose group, AD + SSS medium‐dose group, and AD + SSS high‐dose group. All animal experiments adhered to the guidelines set forth by the National Academy of Sciences of the National Institutes of Health (NIH) and were approved by the Animal Ethical Committee of Jinan University.

### 2.5 Hippocampal Stereotaxic Injection

The mice were anesthetized by inward breath of 2% isoflurane. The Alzheimer's disease models were created through the intracranial ventricular infusion of Aβ_1‐42_(#24224, Anaspec). Aβ_1‐42_ oligomers were prepared using a protocol in accordance with our previous study [24]. Briefly, Aβ_1‐42_ peptides treated with hexafluoroisopropanol (HFIP) were resuspended in dimethylsulfoxide (DMSO). To form oligopeptide, the peptide was diluted to a 100 μM concentration using Ham's F12 medium and subsequently incubated at a temperature of 4°C for 24 h. After a 10‐min centrifugation at 14,000 g, the supernatant was collected and stored. Each mouse brain received two stereotaxic injections at positions symmetrically defined as: anterior‐posterior, −2.0 mm; medial‐lateral, ±1.5 mm; dorsal‐ventral, 2.0 mm. Curtly, 1.5 μL Aβ_1‐42_ oligomers or an equivalent volume of vehicle control was administered bilaterally into the hippocampal region using stereotactic infusion (at 0.5 μL/min) and then left for 10 min to work with dispersion. The microinjector was gradually raised over a time of 2 min.

### 2.6 Behavior Tests

#### 2.6.1 Y Maze

The Y‐maze apparatus was formed with three white obscure plexiglass arms. The size of each arm was 40 cm × 20 cm × 10 cm. The mice were placed at the junction of a Y‐shaped apparatus and granted the freedom to explore the three arms for 8 min. Arm entries and the order of entries were determined from recorded video. The spontaneous alternations in the total arena were recorded.

#### 2.6.2 Novel Object Recognition

On the day prior to the commencement of behavioral tests, mice were accustomed to the apparatus for 10 min daily. The testing method comprised a preparation stage and an inclination test after a postponement of 1 h. For the training phase, a pair of identically shaped, sized, and colored items was positioned along the two‐thirds mark of the box's base diagonal. Each mouse was placed inside the device confronting the same side for a 5‐min adaptation stage. During the test stage, mice were exposed to an identical copy of the object used during the training phase, along with a novel object characterized by distinct shapes and colors. During the initial stages, every object was meticulously cleaned to ensure that olfactory signals wouldn't compromise the results. Analysis was performed by measuring the variation in the time taken to explore the novel object as opposed to the original item, that is, the recognition index: the ratio of duration allocated to investigating new items to the overall duration spent on both new and familiar items. The entire experiment was recorded and analyzed using the EthoVision XT v.14 system (Noldus, Netherlands).

#### 2.6.3 Morris Water Maze

The MWM technique was executed as recently portrayed [25]. The experimental site was a circular pool with a diameter stretching 150 cm, sprinkled with water containing titanium dioxide that was 30 cm deep and maintained at a temperature of 20°C ± 1°C. A white platform, 6 cm in height, was positioned 1 cm below the water surface in the center of a designated quadrant. During the 5‐day training period, mice were expected to stand on the secret submerged platform in three trials outside the quadrant containing the platform. When a mouse was unable to detect the concealed platform, it was directed toward it, where it remained for 30s. Mice had to voyage the pool without the platform for 1 min on the last day of the probe test. The entire experiment was recorded and analyzed using the EthoVision XT v.14 system.

### 2.7 Immunofluorescence Staining

Brain tissue was fixed in 4% PFA for 24 h for later preparation of sections for immunohistochemical analysis. Then the tissues were embedded in an optimal cutting temperature (OCT) solution and frozen following incubation in 30% sucrose for 48 h. Sections (10 μm) were stored at −20°C until required for immunohistochemistry. For assessment of Aβ and Iba1^+^ microglia, sections were incubated in a blocking buffer (phosphate‐buffered saline plus 0.3% Triton X‐100 containing 3% BSA) for 1 h at room temperature (RT). Then, the slices were stained with primary antibodies (anti‐β‐amyloid, 1:1000, # 803001, BioLegend; anti‐Iba‐1, 1:1000, #019–19,741, FUJIFILM Wako) overnight at 4°C and afterwards stained with anti‐rabbit IgG Fab2 Alexa Flour 488(1:1000, 4412 s, CST) and anti‐rabbit IgG Fab2Alxa Alexa Flour 594(1:1000, 8889 s, CST). Images were captured utilizing a laser confocal microscope (Leica Microsystems, Germany) and analyzed using ImageJ software.

### 2.8 Golgi‐Cox Staining and Analysis

The staining procedure was performed following the guidelines provided with the FD Rapid Golgi Stain Kit (#PK401). To summarize, the brain samples were immersed in the staining solution for 2 weeks before being moved to Solution C for 3 days. Subsequently, tissues after dehydration were sectioned into 100‐μm‐thick coronal slices using a cryostat (VT1000S from Leica) and then subjected to a dehydration process. Microscopic images of hippocampal sections were acquired using an Olympus BX53 microscope. For the analysis of dendritic spines, we utilized bright field images at a 40× magnification. We focused on dendrites that were more than 50 μm away from the cell body for spine density calculations. The quantification of dendritic spine density was facilitated by the ImageJ software.

### 2.9 Transmission Electron Microscopy Analysis

Pieces at a size of 1 mm^3^ thick blocks of tissues obtained from the mice hippocampus were chopped up after post‐fixation with a 2.5% glutaraldehyde solution at 4°C overnight. All specimens were subsequently immersed in 1% osmium tetroxide, encased in a mixture of Epon 812 resin and acetone, and securely set in epoxy resin. The tissue blocks were subsequently cut into 50‐nm segments and treated with a 2% solution of uranyl acetate, followed by the application of lead citrate for staining purposes. Synaptic and mitochondrial microstructures were viewed using a Hitachi HT7800 transmission electron microscope (TEM) (Hitachi Co. Ltd., Tokyo, Japan).

### 2.10 UHPLC–MS/MS Analysis

A mixture was prepared by combining 100 μL of serum with 400 μL of a solvent blend (with a ratio of acetonitrile to methanol being 1:1 by volume) that also contained an internal standard at a concentration of 0.02 mg/mL, specifically l‐2‐chlorophenylalanine. The sample was vortexed and sonicated at low temperature for a duration of 30 min, followed by refrigeration at −20°C for 30 min. The supernatants were removed, and the samples were blown dry under nitrogen after centrifugation for 15 min. The precipitates were resolubilized and subjected to low‐temperature sonication for a duration of 5 min, which was then followed by a centrifugation process at 13,000 g and a temperature of 4°C for a period of 10 min. Subsequently, the supernatant was moved to test vials for LC–MS/MS examination. The serum metabolites were differentiated using the Metware Cloud, a free online platform for data analysis (https://cloud.metware.cn).

### 2.11 RNA Sequencing Analysis

The RNA‐seq analysis was conducted using total RNA isolated from the hippocampus. The construction of cDNA libraries and subsequent sequencing were carried out by Novogene Biotechnology Co. (Beijing, China). Excellent peruses were adjusted to the mouse reference genome using Bowtie2. Articulation overflow and variations for every one of the genes were standardized to pieces per kilobase of record per million planned peruses (FPKM) utilizing RNA‐seq by Expectation Maximization (RSEM). We then identified genes with significant expression differences across samples and conducted clustering and functional annotation analyses. Genes with the |log_2_FC| of ≥ 2 and a false discovery rate (FDR) of < 0.05 were deemed statistically significant. Pathways that were notably enriched with DEGs were annotated in the KEGG (Kyoto Encyclopedia of Genes and Genomes) database.

### 2.12 Western Blotting

The mouse brain hippocampus tissues were homogenized in RIPA lysis buffer (#89900, Thermo Fisher Scientific, USA) containing protease inhibitor (Servicebio, China) on ice for 30 min and centrifuged at 14000 × g for 20 min at 4°C. The experiment was performed according to our previous experimental steps [26]. The supernatants were gathered and applied to the BCA assay (#P0010, Beyotime, China) to judge protein concentration and then separated by 10% and 12% sodium dodecyl sulfate–polyacrylamide gel electrophoresis (SDS–PAGE) followed by transferring the proteins from the gels to polyvinylidene difluoride (PVDF) membrane (Millipore, Germany) and blocking the membranes with 5% skim milk. The membranes were exposed to the primary antibodies for an extended period at a temperature of 4°C: anti‐PSD95 (1:1000, #3409, CST), anti‐Parkin (1:1000, #4211, CST), anti‐LC3A/B (1:1000, #12741, CST), anti‐SQSTM1/P62 (1:1000, #5114, CST), anti‐Beclin1(1:1000, #3495, CST), anti‐GAPDH(1:5000,#60, 004‐1. Proteintech) and β‐actin (1:5000, #380624, ZEN BIO). Following a thorough wash with 1× TBST and a subsequent incubation with secondary antibodies for an hour, the membranes were treated with a fluorescent solution. Images were obtained using the ChemiDoc MP Imaging System (Bio‐Rad, USA). The densities of the bands were quantified by using ImageJ, while graphs were generated by GraphPad Prism 8 software.

### 2.13 Statistical Analysis

Experimental data were statistically analyzed using GraphPad Prism 9 software, with results presented as mean ± S.E.M. Each wet‐lab experiment was conducted in a minimum of three independent trials. Statistical significance was determined at *p* < 0.05. Group variations were assessed using one‐way ANOVA followed by Šidák's multiple comparisons test or two‐way ANOVA followed by Tukey's or Dunnett's multiple comparisons test for multiple groups, assuming a normal distribution. All data were tested for normality by Shapiro–Wilk. The Kruskal‐Wallis test was employed to analyze data that did not follow a normal distribution.

## 3 Results

### 3.1 The Components of Sanshen San and Their Interaction With AD

**FIGURE 1:**
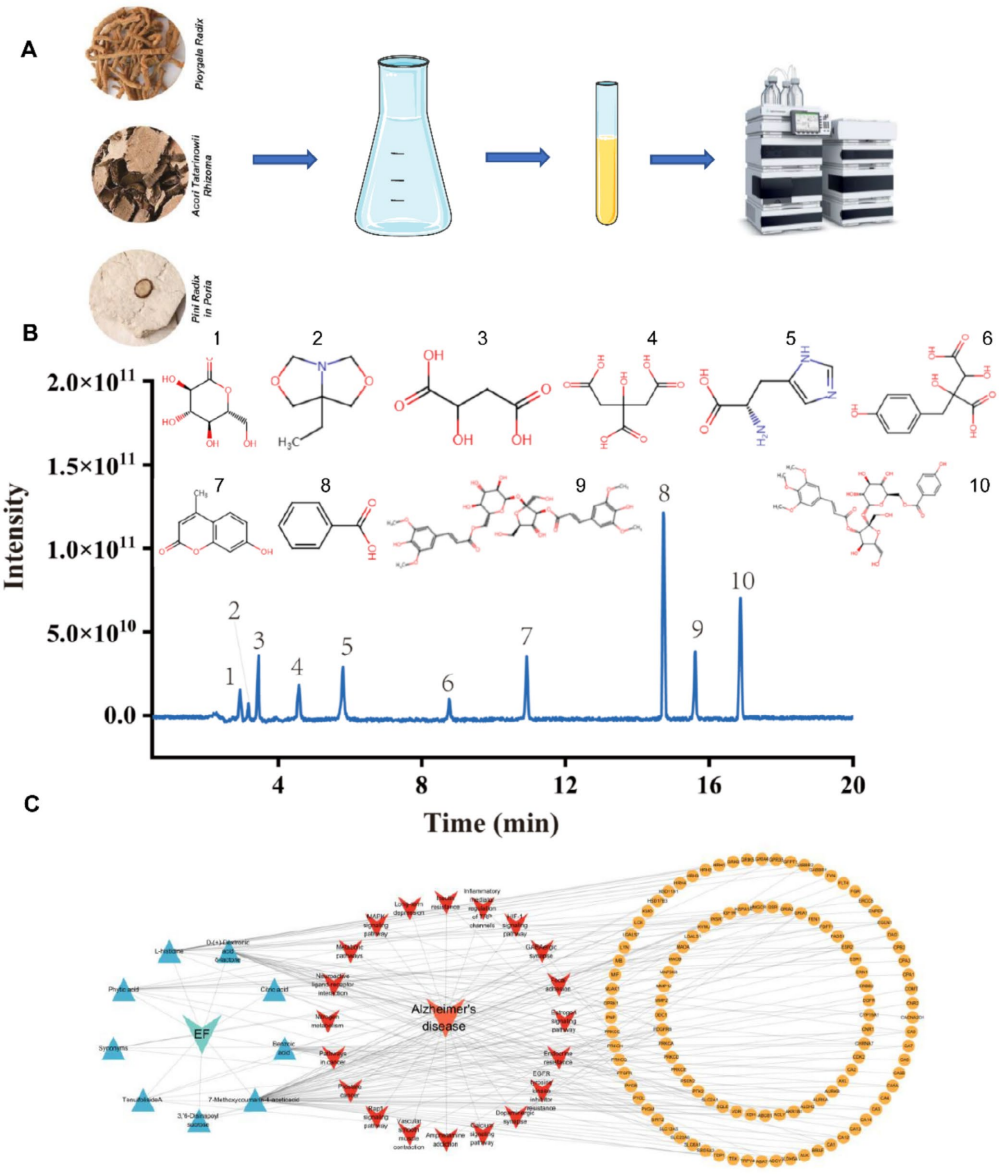
The components of Sanshensan and their interaction with Alzheimer's disease. (A) Composition of SSS, which is a mix of three traditional Chinese herbs, as well as a diagram of the extraction strategy; (B)Total Ion chromatograms and main components; (C) pC‐pT‐pP network of SSS components in the treatment of AD. The blue, orange, and red nodes represent active compounds, target genes, and pathways, respectively.

SSS was made up of *Polygala Radix*, *Pini Radix in Poria*, and *Acori Tatarinowii Rhizoma* (Figure 1A). UHPLC–MS/MS was used for the identification of the major chemical constituents of SSS for the confirmation of the chemical profile of SSS for quality assurance purposes. Hundreds of compounds were detected, with 10 major compounds identified overall from SSS (Figure 1B), as detailed in Table 1. The substances were D‐(+), ‐Dextronic acid δ‐lactone, 7a‐Ethyltetrahydro‐1H‐oxazolo[3,4‐c]oxazole, Synonyms, Citric acid, L‐histidine, Phytic acid, 7‐Methoxycoumarin‐4‐acetic acid, Benzoic acid, 3,’6‐Disinapoyl sucrose, and TenuifolisideA (Table 1). We utilized the network analysis to discover the feasible mechanism of SSS treating AD. KEGG pathway enrichment analysis revealed that the primary involvement of these targets was metabolism pathways, neuroactive ligand‐receptor interaction, and dopaminergic synapse (Figure 1C).

**TABLE 1:** List of 10 major compounds by UHPLC–MS/MS.

No	Compounds	Formula	Mw	Retention time
1	D‐(+)‐Dextronic acid δ‐lactone	C_6_H_10_O_6_	178	2.95
2	7a‐Ethyltetrahydro‐1H‐oxazolo[3,4‐c]oxazole	C_7_H_13_NO_2_	143	3.18
3	Synonyms	C_4_H_6_O_5_	134	3.45
4	Citric acid	C_6_H_8_O_7_	192	4.58
5	L‐histidine	C_6_H_9_N_3_O_2_	155	5.79
6	Phytic acid	C_11_H_12_O_7_	256	8.77
7	7‐Methoxycoumarin‐4‐aceticacid	C_10_H_8_O_3_	176	10.91
8	Benzoic acid	C_7_H_6_O_2_	122	14.74
9	3,’6‐Disinapoyl sucrose	C_34_H_42_O_19_	754	15.61
10	TenuifolisideA	C_31_H_38_O_17_	682	16.87

### 3.2 Sanshen San Restrains Learning and Memory Deficits in the AD‐Like Model of Acute Aβ_1‐42_ Injection

**FIGURE 2:**
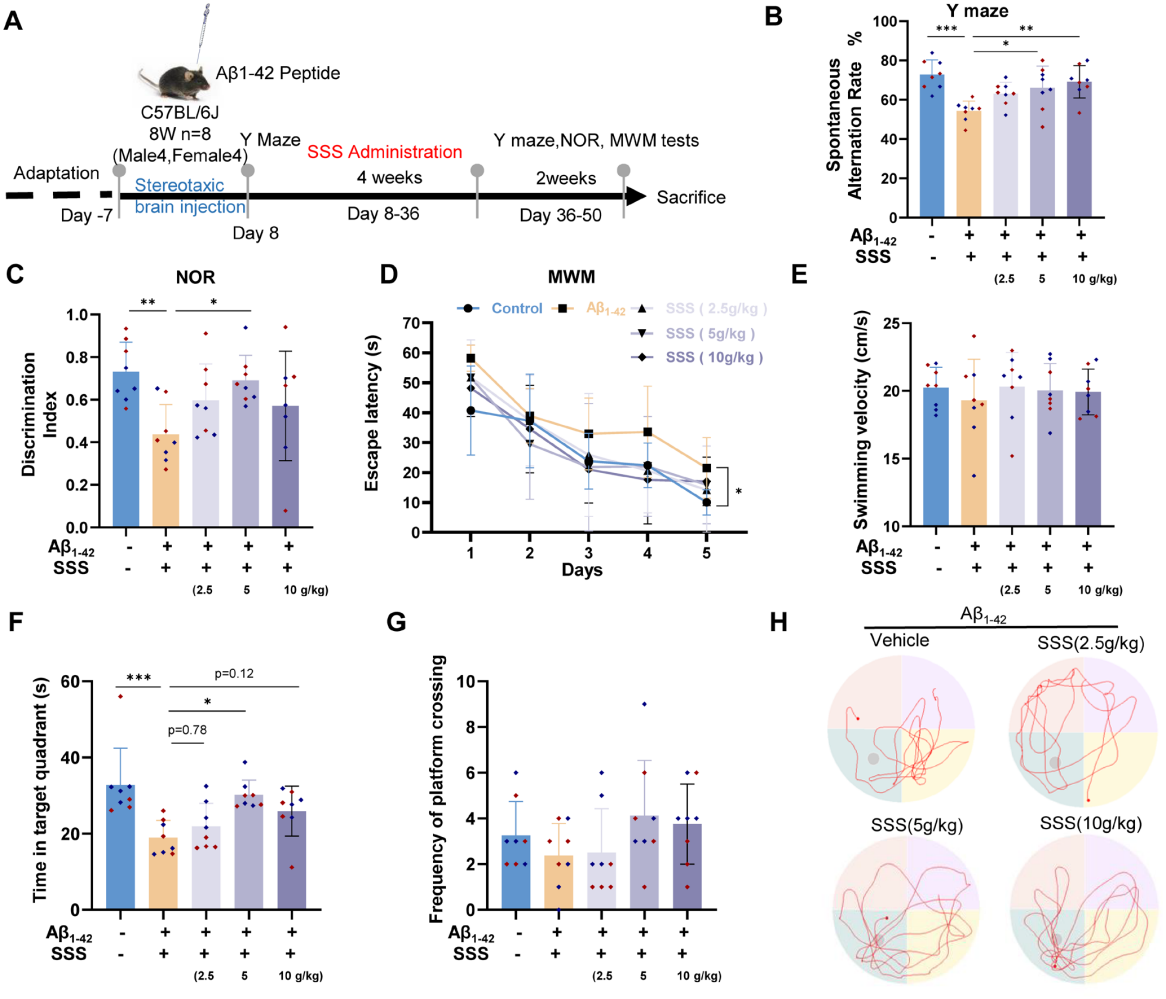
Sanshen San restrains learning and memory deficits in the AD‐like model of acute Aβ_1‐42_ injection. (A) The graph presents the experimental scheme. Aβ_1‐42_ or vehicle was injected into the hippocampus of C57BL/6J mice. (B)Y maze test. (C) Novel object recognition test. (D) Escape latency in the MWM test during the 5‐day training and learning regimen. Two‐way ANOVA followed by Student–Newman–Keuls post hoc analysis (time effect: F4,200 = 39.77, *p* < 0.001); group effect: F4,200 = 3.401, *p* = 0.0102); group × time effect: F16,200 = 0.6248, *p* = 0.8617). (E) Average swimming velocity. (F) Time in the target quadrant. (G) Frequency of crossing the platform. (H) Representative traces in MWM tests. The blue dots represent male mice, and the red dots represent female mice. The data were mean ± SEM (*n* = 8 per group). *p < 0.05; **p < 0.01; ***p < 0.001.

The experimental schedule is shown in Figure 2A. The mice were subjected to the Y maze to evaluate their cognitive function to ensure the model's feasibility 1 week after an injection of Aβ_1‐42_. After a 4‐week treatment, the mice were placed to explore freely without food in the Y‐maze test, and the SSS‐treated mice showed a prolonged period of exploration in the target arm compared to vehicle‐treated AD‐like mice, medium‐dose, and high‐dose groups especially (*p* < 0.05 & *p* < 0.01; Figure 2B). We then found the discrimination index of mice in the medium‐dose‐treated group for novel objects increased significantly (*p* < 0.05, Figure 2C). To assess hippocampus‐dependent spatial learning and memory, we also performed the MWM test. The model group showed longer escape latency during MWM training (Figure 2D). In addition, at the same swimming speeds, fewer mice in the model group were able to successfully locate the platform compared to the control group. The velocity of the probe during the test remained consistent, showing no variation (Figure 2E). The medium‐dosage group mice spent more time in the target quadrant than the model group (*p* < 0.05, Figure 2F). There was a trend toward an increase in the frequency of target crossings in SSS‐treated mice compared to the control group, although this was not statistically significant (Figure 2G). The SSS formula reduced the degree of memory impairment in the mouse models, with the medium concentration grouping performing better(Figure 2G,H).

### 3.3 Sanshen San Inhibited Learning and Memory Deficits in 5xFAD Mice

**FIGURE 3:**
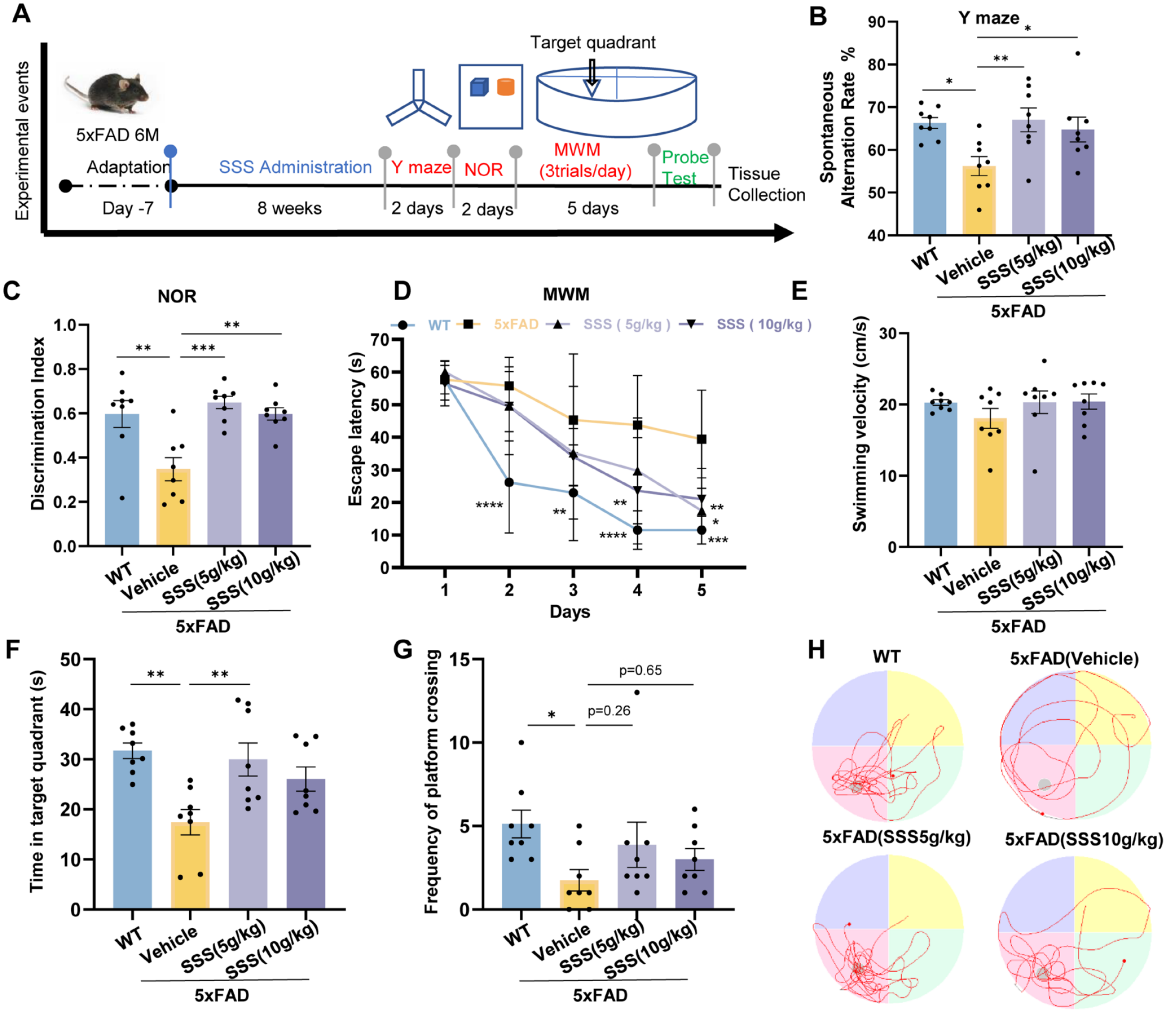
Sanshen San inhibited learning and memory deficits in 5×FAD mice. (A) The graph shows the experimental scheme. (B) Y maze test. (C) Novel object recognition test. (D) Escape latency in MWM test during the 5‐day training and learning regimen. Two‐way ANOVA followed by Student–Newman–Keuls post hoc analysis (group effect: F3,135 = 20.44, *p* < 0.001); time effect: F4,135 = 39.66, *p* < 0.001); group × time effect: F12,135 = 2.063, *p* = 0.023). (E) Swimming velocity. (F) Duration time in the target quadrant. (G) Frequency of platform crossing. (H) Representative trajectories in MWM tests. The data were mean ± SEM (*n* = 8 per group). *p < 0.05; **p < 0.01; ***p < 0.001.

Based on the better performance of the medium‐dose group in the AD‐like mice, we selected the medium and high doses for the following trial. We chose medium and high dosage SSS to recognize Alzheimer's disease‐mice 5xFAD. The experimental procedure is demonstrated in Figure 3A. Y‐maze and NOR tests are widely used in the neurosciences for the assessment of learning and memory skills. Utilizing the Y‐Maze evaluation, we found that the spontaneous alternation rate of the AD mice was relatively lower than that of the wild‐type mice. (*p* < 0.05, Figure 3B) while SSS‐treated mice showed better spatial memory. During the NOR test period, the discrimination index of mice in the SSS‐treated group for novel objects increased significantly (*p* < 0.001& *p* < 0.01, Figure 3C). Finally, assessment of cognitive function was conducted by taking the mice to a MWM test. In the navigation assay, vehicle‐treated 5xFAD mice had significantly longer escape latencies than WT mice, suggesting a behavioral abnormality (*p* < 0.01, Figure 3D). While SSS medium‐dose treatment decreased the escape latency significantly. In the spacecraft test, AD mice spent less time in the platform area, navigating primarily around the tank's periphery (*p* < 0.01, Figure 3F). SSS medium‐dose treatment markedly spent more time in the target quadrant in 5xFAD mice (*p* < 0.01, Figure 3F) and this group showed a tendency to increase the frequency of platform crossings, suggesting an improvement in cognitive performance compared to the AD mice (*p* = 0.26, Figure 3G). There were no marked disparities in the speed of the swimmers between the groups during the probe test (Figure 3E). These results suggest that SSS treatment effectively enhanced cognitive performance in AD mice. The swimming paths of representative mice are presented in Figure 3H.

### 3.4 Sanshen San Impedes Aβ Pathology and Spine Loss in 5xFAD Mice

**FIGURE 4:**
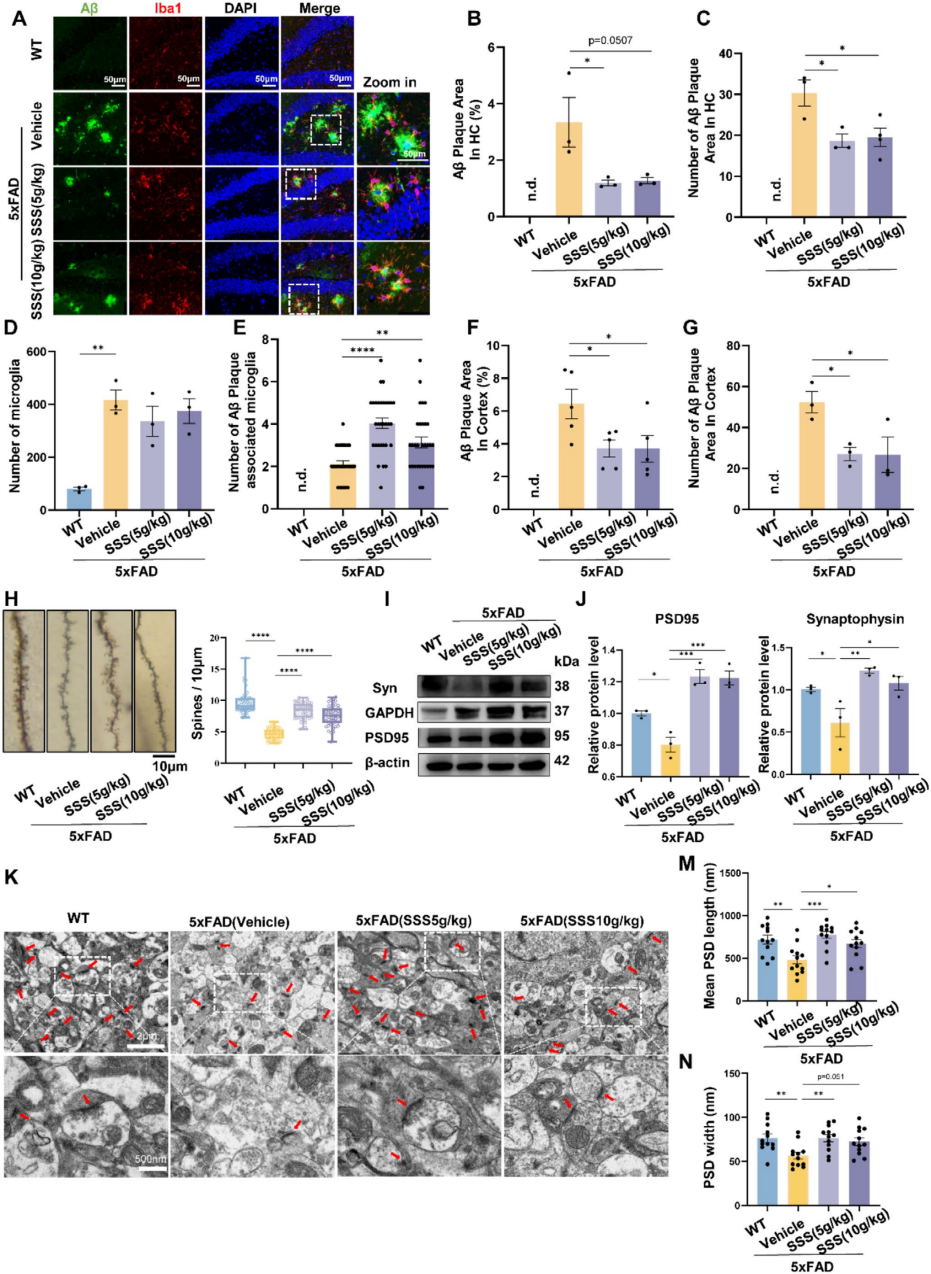
Sanshen San impedes Aβ pathology and spine loss in 5xFAD mice. (A)Representative images of Aβ plaques (6E10) in DG areas of the hippocampus of 5xFAD and SSS‐treated 5×FAD mice. Scale bar =50 μm. (B, C) The bar graph represents the quantification of Aβ plaque area in the hippocampus. (D, E) Number of microglia. (F, G) Percentage of Aβ plaque area measured in the cortex. For (A) to (G), *n* = 3 mice per group, 3–5 slices each tissue). (H) Golgi‐Cox staining for the measurement of synapse density in the hippocampal neurons. High magnification imaging shows the spine density on basal dendrites per 10 μm. Scale bar = 10 μm. WT, 9.61 ± 0.25; AD, 4.67 ± 0.13; SSS‐M,8.42 ± 0.17; SSS‐H,7.56 ± 0.22. *n* = 4 mice per group; *n* = 47 to 50 dendrites per group were counted. (I) Representative Western blot data and (J) quantification of Synaptophysin and PSD95 proteins in the hippocampus. (K) Representative TEM images of synaptic structures. Scale bar = 2 μm/500 nm. (M, N) Quantification of postsynaptic density length and width. Data were presented as mean ± SEM. *p < 0.05; **p < 0.01; ***p < 0.001; ****p < 0.0001.

We conducted immunofluorescence staining to determine whether the Sanshen San formula enhances the behaviors of 5xFAD mice through a reduction in Aβ deposits within their brains. Our research indicates that the application of SSS notably reduced both the quantity and extent of Aβ plaques in the hippocampus and in the cortex of 5xFAD mice (Figure 4A–G & Figure S1). The cerebrum's immune defense is carried out by specialized cells known as microglia. They are the basic cell type for clearing other microorganisms, including Aβ. Simultaneously, we evaluated the regulation of microglial activation in regions of the hippocampus and cortex to investigate the mechanism of Aβ clearance by SSS. Visual inspection of the zoomed‐in enlarged images in the immunofluorescence staining demonstrated that SSS promoted the phagocytic activity of Aβ by microglia (Figure 4A). To assess the neuroprotective effects of SSS on synaptic damage in 5xFAD mice, we conducted an analysis of the hippocampal synaptic structure, dendritic spine density, and detected synaptic protein levels. We examined hippocampal synapse ultrastructure and surveyed postsynaptic density length and width using Image J software. Decreased synaptic counts were observed and were reversed by SSS. The application of Golgi‐Cox staining techniques disclosed a marked decrease in the density of dendritic spines in hippocampal neurons of 5xFAD mice when juxtaposed with their wild‐type counterparts. Conversely, a marked rise in the density of dendritic spines was observed in the hippocampal neurons of the SSS cohort (*p* < 0.0001, Figure 4H). Western blotting was then used to determine the expression of synaptic marker proteins—synaptophysin and PSD95. In the hippocampal region of 5xFAD mice, the levels of PSD95 protein were considerably lower when compared to the levels observed in the control group (Figure 4I). In contrast to the AD group, there was a notable elevation in the levels of PSD95 expression within the hippocampus for the SSS group (*p* < 0.001, Figure 4I). As depicted in Figure 4J, there is a notable decrease in both the length and breadth of the postsynaptic density within the hippocampus in the AD cohort when contrasted with the control group exhibiting wild‐type characteristics. There was a notable augmentation in both the dimensions of the postsynaptic density within the hippocampal region for the SSS groups in contrast to the AD group (*p* < 0.01 & *p* < 0.05, Figure 4J). These results illustrated that SSS could promote the amelioration of synaptic and cognitive impairment in AD.

### 3.5 Sanshen San Treatment Rescues Mitochondrial Function and Enhances Autophagy in 5xFAD Mice

**FIGURE 5:**
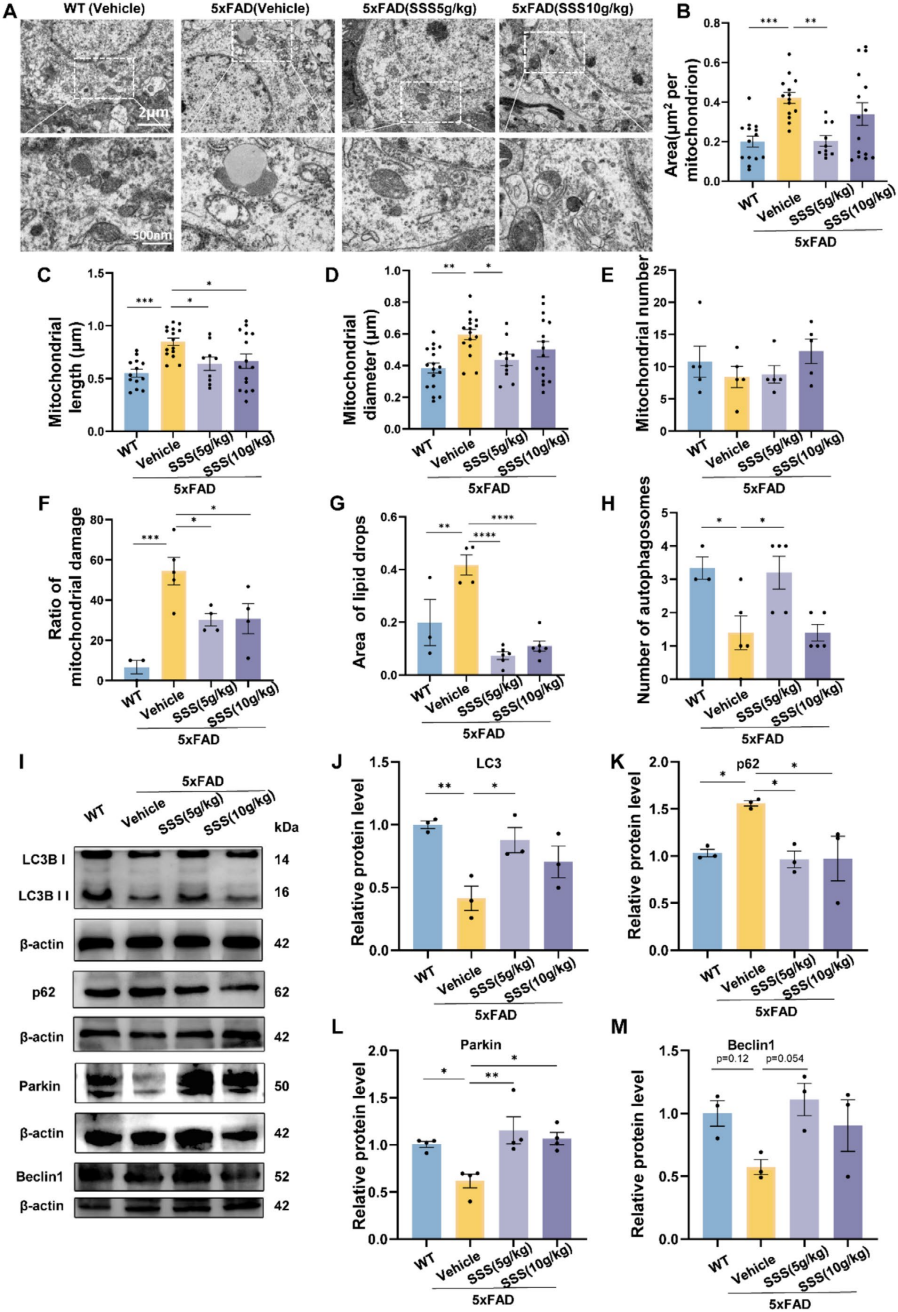
Sanshen San inhibits mitochondrial damage and enhances autophagy in 5×FAD mice. (A) Representative TEM images of mitochondrial structures. (B–H) Quantification of mitochondrial area, mitochondrial length, mitochondrial diameter, mitochondrial number, ratio of mitochondrial damage, lipid drop area, and autophagosomes. (I–M) Representative band diagram and quantification of LC3, p62, Parkin, and beclin1 proteins in the hippocampus. Data were presented as mean ± SEM. *p < 0.05; **p < 0.01; ***p < 0.001; ****p < 0.0001.

Mitochondria play a crucial role in brain neurons. Significant changes in mitochondrial morphology were found in the different groups by TEM analysis (Figure 5A–H). The vehicle‐treated 5xFAD mice showed increased mitochondrial area (*p* < 0.001) and diameter (*p* < 0.001) compared to WT controls. SSS treatment (5 and 10 g/kg) effectively normalized these parameters (*p* < 0.05, Figure 5B–D). The ratio of damaged mitochondria was markedly elevated in 5xFAD mice that received the vehicle treatment compared to WT mice (*p* < 0.001), while SSS treatment significantly reduced this ratio (*p* < 0.05, Figure 5F). Similarly, the area of lipid drops and the number of autophagosomes were significantly altered in 5xFAD mice and restored by SSS treatment (*p* < 0.05, Figure 5G,H). The process of Western blotting was utilized to investigate proteins associated with autophagy (Figure 5I). LC3 protein expression level was considerably lower in 5xFAD mice treated with the vehicle compared to those of the wild‐type controls (*p* < 0.01), while SSS treatment restored LC3 expression (*p* < 0.05, Figure 5J). Conversely, p62 levels were elevated in 5xFAD mice (*p* < 0.05) and normalized by SSS treatment (Figure 5K). Additionally, Parkin expression was decreased in vehicle‐treated 5xFAD mice (*p* < 0.05) but was restored following SSS treatment (*p* < 0.01, Figure 5L). Beclin1 showed a similar trend, although the changes did not reach statistical significance (*p* > 0.05, Figure 5M). These results suggest that Sanshen San treatment effectively improves mitochondrial function and enhances autophagy in 5xFAD mice, potentially contributing to its therapeutic effects.

### 3.6 RNA‐Seq and Metabolomics Analysis Reveals That Sanshen San Restores the Neurotransmitter Function Between Synapses in 5xFAD Mice

**FIGURE 6:**
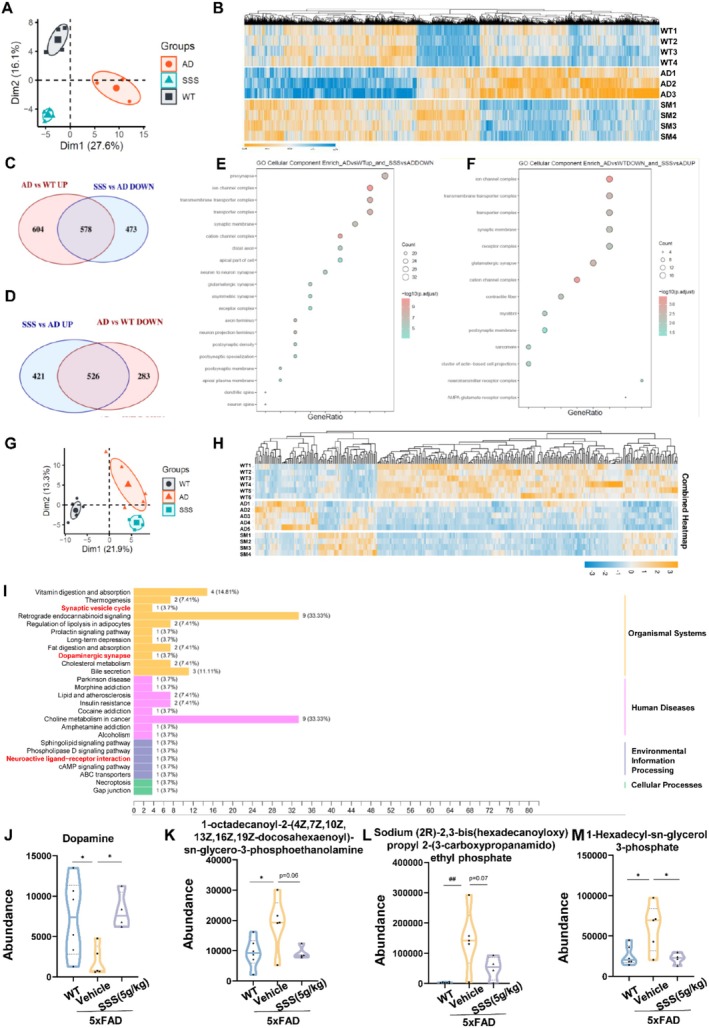
RNA‐seq and metabolomics analysis revealed that Sanshen San restored the neurotransmitter function between synapses in 5×FAD mice. (A) PCoA analysis; (B) Heatmap showing the differentially expressed genes in the cortex area of WT, AD, and SSS‐treated mice; (C, D) Number of differentially expressed genes; (E, F) Top 10 GO cellular component enrichment of differentially expressed genes from (C, D); (G) PCoA analysis; (H) Heatmap from mice serum metabolism; (I) KEGG classification of differential metabolites between SSS and AD groups; (J–M) Representative metabolites connecting to synapses differentially expressed.

RNA sequencing and metabolomic analyses revealed distinct molecular signatures among WT, 5xFAD, and SSS‐treated groups. Principal component analysis (PCA) demonstrated clear separation between these groups, indicating significant transcriptional and metabolic alterations (Figure 6A,G). Hierarchical clustering analysis of differentially expressed genes (DEGs) revealed distinct transcriptional patterns among WT, 5xFAD, and SSS‐treated groups (Figure 6B). The heatmap visualization demonstrated three major gene clusters with distinct expression patterns. Differential expression analysis identified 604 genes uniquely dysregulated in 5xFAD mice compared to WT controls, while 473 genes were differentially expressed between SSS‐treated and untreated 5xFAD mice (Figure 6C). The Venn diagram analysis revealed 536 overlapping genes between AD vs. WT DOWN and SSS vs. AD UP comparisons, suggesting SSS treatment partially restored the expression of genes downregulated in 5xFAD mice (Figure 6D). Gene Ontology (GO) analysis has uncovered a substantial overrepresentation of biological processes concerning neurotransmitter function, synaptic transmission, and cellular component organization (Figure 6E,F). GO biological process analysis (Figure 6A,D) revealed enrichment in neuronal development, synaptic transmission, and neurotransmitter regulation. Molecular function analysis (Figure S2A,E) showed significant changes in ion channel activity and neurotransmitter receptor function. KEGG pathway analysis (Figure S2C,F) identified alterations in key signaling pathways related to synaptic plasticity and neurotransmitter systems. The heatmap visualization demonstrated that SSS treatment substantially normalized the expression patterns of these pathway‐related genes (Figure S2B,H). Metabolomic profiling identified significant alterations in several key pathways (Figure 6I), with particularly notable changes in neurotransmitter systems. Specifically, dopamine levels were significantly decreased in vehicle‐treated 5xFAD mice compared to WT controls (*p* < 0.05), while SSS treatment effectively restored dopamine levels (Figure 6J). Additionally, several metabolites associated with neural function showed similar patterns of restoration following SSS treatment (Figure 6K–M).

These findings suggest that SSS treatment can effectively normalize both transcriptional and metabolic alterations in 5xFAD mice, particularly those related to neurotransmitter function and synaptic transmission, providing molecular evidence for its therapeutic potential in AD.

## 4 Discussion

Our comprehensive study demonstrates that SSS exhibits significant therapeutic potential in AD through multiple mechanisms. SSS is composed of 3 herbs, the most frequently used herbs, which give rise to the complex chemical constituents and may target a variety of pathways. It has been reported that *Polygala Radix* extract may have a protective effect against axonal degeneration and memory impairment through inhibition of endocytosis [27]. *Pini Radix in Poria*, a parasitic herb used in traditional Chinese medicine mainly for palpitations and amnesia, attenuated BaCl2‐induced cardiac arrhythmia damage by suppressing RyR2 expression and reducing adrenaline and cAMP through the adrenergic signaling pathway [28]. *Acori Tatarinowii Rhizoma* treatment on 3xTg mice in Fu's study improved the basic cognitive capacity of AD mice and the excessive phosphorylation of Tau protein was decreased indicating that *Acori Tatarinowii Rhizoma* could safeguard the myelin sheath against degeneration in AD [29]. The investigation spanned from component analysis to behavioral studies, and from cellular pathology to molecular mechanisms, providing multi‐level evidence for SSS's efficacy. First, our chemical profiling of SSS identified 10 principal bioactive compounds, with network analysis revealing their potential mechanisms primarily involving metabolism pathways, neuroactive ligand‐receptor interaction, and dopaminergic synapse function. This finding provided the molecular basis for SSS's therapeutic effects.

The behavioral studies in both acute Aβ_1‐42_ injection model and 5xFAD mice demonstrated SSS's robust cognitive‐enhancing effects. In this study, the dosing regimen was selected based on the clinical human dosage, with reference to the dosages used in animal experiments for similar traditional Chinese medicine formulations, such as Kaixin San, to ensure that the selected dose is within the safety range and has pharmacological activity [30]. Particularly, the medium‐dose treatment showed superior efficacy in improving spatial memory and learning ability, as evidenced by Y‐maze, novel object recognition, and Morris water maze tests. These behavioral improvements suggest SSS's potential in alleviating AD‐related cognitive deficits. It is worth mentioning that both male and female mice were used in acute Aβ1‐42 injection model while we did not find gender differences in SSS‐induced benefits. Much of the current research assessing sex differences in neurodegeneration is either descriptive. To generate reliable data and advance the development of effective therapeutics, it is essential to design experiments with adequately powered sample sizes for both male and female subjects [31].

Extremely enormous data has been reported that the formation and expansion of Aβ plaques are facilitated by the improper folding, autonomous aggregation, and subsequent dissemination of Aβ proteins, contributing to the toxic impact on the brain of AD patients [32]. Therefore, focusing on Aβ has been an essential technique in creating DMTs for Promotion over the most recent couple of many years [33]. The removal of Aβ is a crucial process that involves numerous essential elements such as proteases, microglial phagocytosis, autophagic‐lysosomal pathways, as well as the transportation across both the blood‐cerebrospinal fluid barrier and the blood–brain barrier [34, 35, 36]. As the innate immune cells of the central nervous system (CNS), microglia, upon activation, engage in the removal of misfolded proteins, cellular debris, and pathogens [37]. This process plays a crucial role in preserving neuronal integrity and reducing neuroinflammation [38]. The engulfment of harmful beta‐amyloid by microglia is crucial for its removal, while mitochondria are increasingly recognized as the central hub of innate immunity [39]. Autophagy, the constitutive turnover of obsolete proteins and organelles, is the major pathway for lysosomal degradation and maintains balance within cellular environments [40]. The process is further triggered by illness and cellular strain to dispose of irregular proteins, clumps, and impaired cellular components [41]. Through swift integration with lysosomes and the breakdown of autolysosome contents by activation of autophagy, healthy neurons effectively dispose of newly generated autophagosomes or amphiboles [42]. To transmit signals effectively, the synapse requires a substantial energy supply. Dysfunctional mitochondria in AD prevent the synapse from doing its job properly, giving rise to reduced neurotransmission and weakened synaptic connections [43]. This results in diminished cognitive function and a decline in memory, which are characteristic symptoms of AD. Meanwhile, mitophagy is the essential mechanism to eliminate damaged mitochondria [44]. Thus, turning around synaptic debilitation is an elective technique for AD treatment [14].

At the cellular level, SSS demonstrated significant neuroprotective effects through multiple mechanisms: Reduction of Aβ pathology: SSS effectively decreased both the number and size of Aβ plaques in the hippocampus and cortex, possibly through enhanced microglial phagocytic activity. Synaptic protection: SSS treatment preserved synaptic structure and function, as evidenced by increased dendritic spine density and restored synaptic protein expression (PSD95 and Synaptophysin). Mitochondrial function improvement: SSS normalized mitochondrial morphology and reduced the damaged mitochondria ratio in 5xFAD mice. Enhancement of autophagy: The treatment restored autophagy‐related protein expression (LC3, p62, Parkin), suggesting improved cellular clearance mechanisms.

Most notably, our integrated transcriptomic and metabolomic analyses revealed that SSS's therapeutic effects are largely mediated through the restoration of neurotransmitter function and synaptic transmission. The RNA‐seq data showed normalized expression of genes involved in synaptic function and neurotransmitter regulation. Following SSS treatment, enrichment analysis of GO cellular components compared to the AD group revealed significant involvement in processes such as glutamatergic synapse, asymmetric synapse, postsynaptic density, and neuron spine. While metabolomic profiling demonstrated restored dopamine levels and other neural‐function‐associated metabolites. KEGG enrichment analysis of differential metabolites highlighted synaptic vesicle cycle, dopaminergic synapse, and neuroactive ligand‐receptor interaction especially. An FP‐CIT PET imaging study clinically investigated that presynaptic dopaminergic deficit might be one of the possible reasons for parkinsonian signs in AD [45]. These molecular findings provide mechanistic insights into how SSS improves cognitive function in AD.

In conclusion, our findings suggest that SSS acts through multiple complementary mechanisms to ameliorate AD pathology, with particular emphasis on restoring synaptic function and neurotransmitter homeostasis. These results not only validate the traditional use of SSS but also provide scientific evidence for its potential as a therapeutic agent in AD treatment.

## 5 Limitation of the Study

Despite the promising results obtained in this study, several limitations should be acknowledged. While we identified multiple bioactive compounds through UHPLC–MS/MS analysis, the precise contribution of each compound to the therapeutic effects and their potential interactions is unclear. Additionally, the long‐term safety profile and potential drug–drug interactions with conventional AD medications were not evaluated in our current study. Furthermore, multiple molecular targets and neurotransmitter systems (e.g., cholinergic, glutamatergic) require further investigation. Finally, the translation of these findings to human patients requires further clinical investigation. These limitations suggest important directions for future research to fully understand the therapeutic potential of SSS in AD treatment.

## Author Contributions

Jun‐Ping Pan, Li Yan, and Jia‐Xu Chen designed the research; Shi‐Quan Chang, Na‐Na Ding, Ya‐Lin Li, Ying Li, and Zi‐Ling Tang performed the research; Shi‐Quan Chang and Jun‐Ping Pan analyzed the data and wrote the manuscript. All authors read and approved the final manuscript.

## Conflicts of Interest

The authors declare no conflicts of interest.

## Supporting information


Appendix S1.

## Data Availability

The datasets used and/or analyzed during the current study are available from the corresponding authors upon reasonable request.
