# Supplementary material for: Sanshen San Formula Hinders Cognitive Function and Pathology in Alzheimer's Disease Through Potentiating the Function of Synapse
Source: CNS Neurosci Ther. 2025 Apr 9;31(4):e70349. doi: 10.1111/cns.70349 (PMC11979623; doi:10.1111/cns.70349)
Supplement: Supplementary file 1 — Appendix S1. [file CNS-31-e70349-s001.docx]

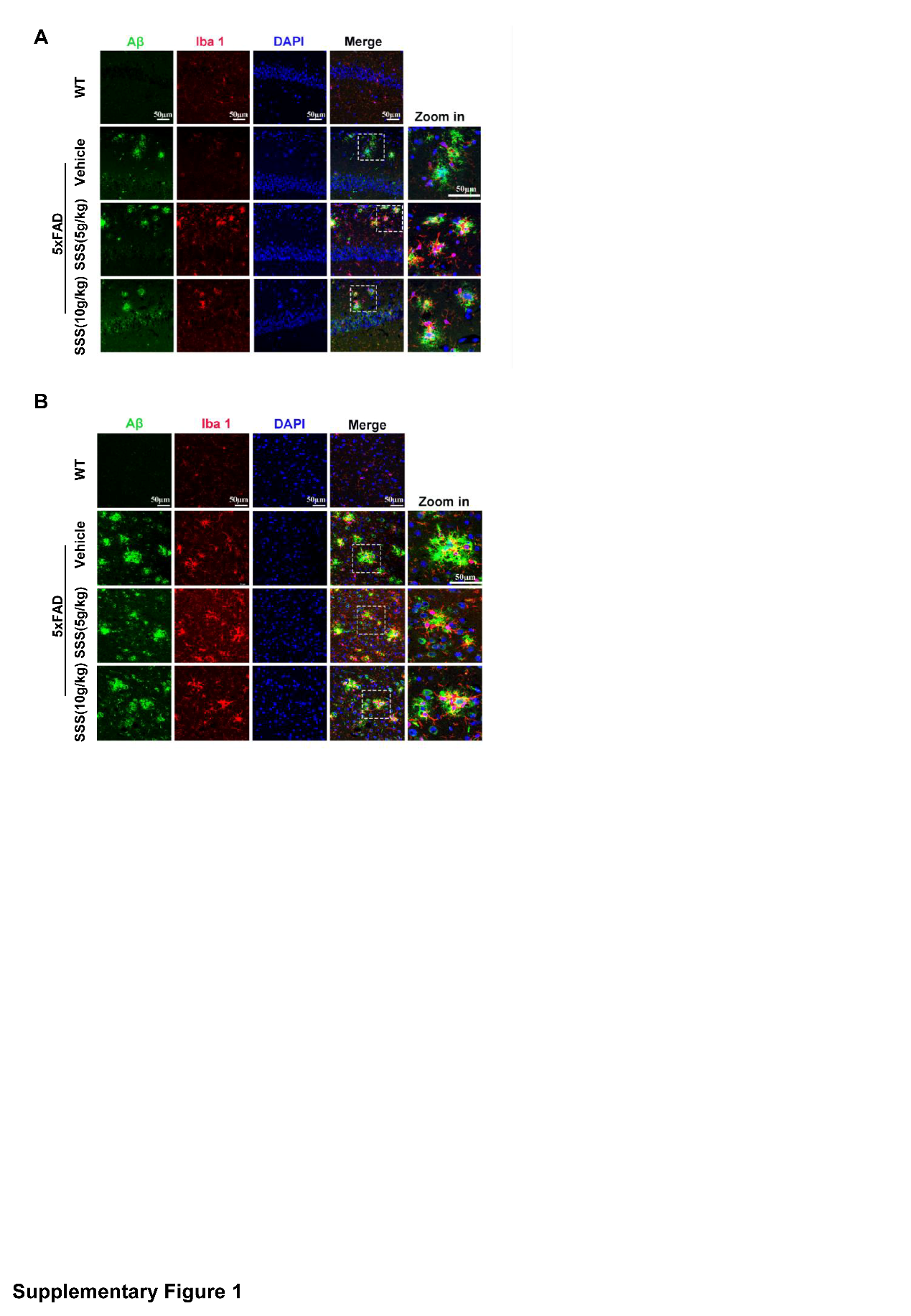


**Supplementary Figure 1**. Representative images of Aβ plaques (6E10) in CA1 areas(A) and cortex areas(B)of the hippocampus of 5xFAD and SSS treated 5xFAD mice. Scale bar corresponds to 50μm.


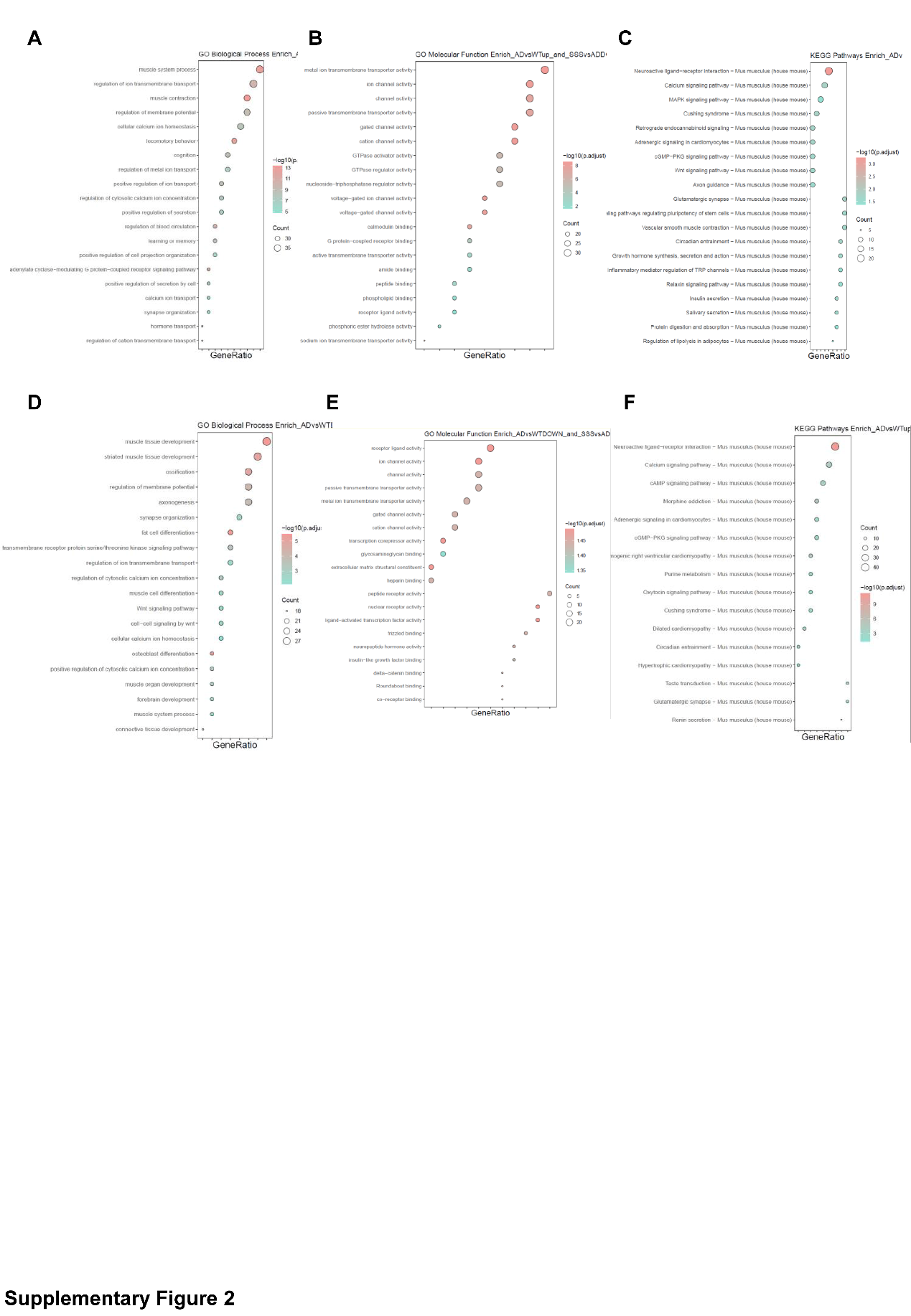


**Supplementary Figure 2**. GO enrichment analysis bubble chart of biological process terms(A) and molecular cellular terms(B);(C) KEGG analysis chart; GO enrichment analysis chart of biological process terms(D) and molecular cellular terms(E);(F) KEGG analysis chart.
